# Metastatic Pure Choriocarcinoma Masquerading in the Gastrointestinal Tract

**DOI:** 10.7759/cureus.64566

**Published:** 2024-07-15

**Authors:** Parvir Aujla, Abdullah S Aleem, Rohit Khanna, Nitish Mittal, John Lyons

**Affiliations:** 1 Internal Medicine, University of Texas Health Science Center at Houston, Houston, USA; 2 Gastroenterology and Hepatology, Scripps Clinic, La Jolla, USA

**Keywords:** intestinal metastasis, dysphagia, upper gastrointestinal bleed, germ cell tumor, pure choriocarcinoma

## Abstract

This study presents a rare case of pure choriocarcinoma (PCC) with metastasis to the gastrointestinal tract in a 52-year-old male with a history of mixed germ cell tumor in remission. Despite negative oncology surveillance imaging, serum marker monitoring, and a recent colonoscopy, the patient presented with new-onset melena and dysphagia, leading to further diagnostic evaluation. Endoscopic examination revealed an ulcerated duodenal mass, and a computer tomography (CT)-guided liver biopsy confirmed metastatic PCC. This case highlights the aggressive nature of PCC and the importance of considering gastrointestinal metastasis in patients with atypical symptoms, even when in apparent remission.

## Introduction

Pure choriocarcinoma (PCC) accounts for less than one percent of the germ cell tumor subtype but is notorious for its aggressive course [1]. Approximately 70% of patients at the time of diagnosis present with metastatic lesions [2]. In the following report, we present the case of a patient with a previous history of mixed germ cell tumor in remission who presented with alarming symptoms of melena. Subsequent endoscopic examination unveiled a bleeding, ulcerated duodenal mass. A liver biopsy ultimately confirmed the presence of metastatic PCC, underscoring the importance of vigilance in recognizing atypical presentations of this aggressive malignancy.

## Case presentation

**Figure 1:**
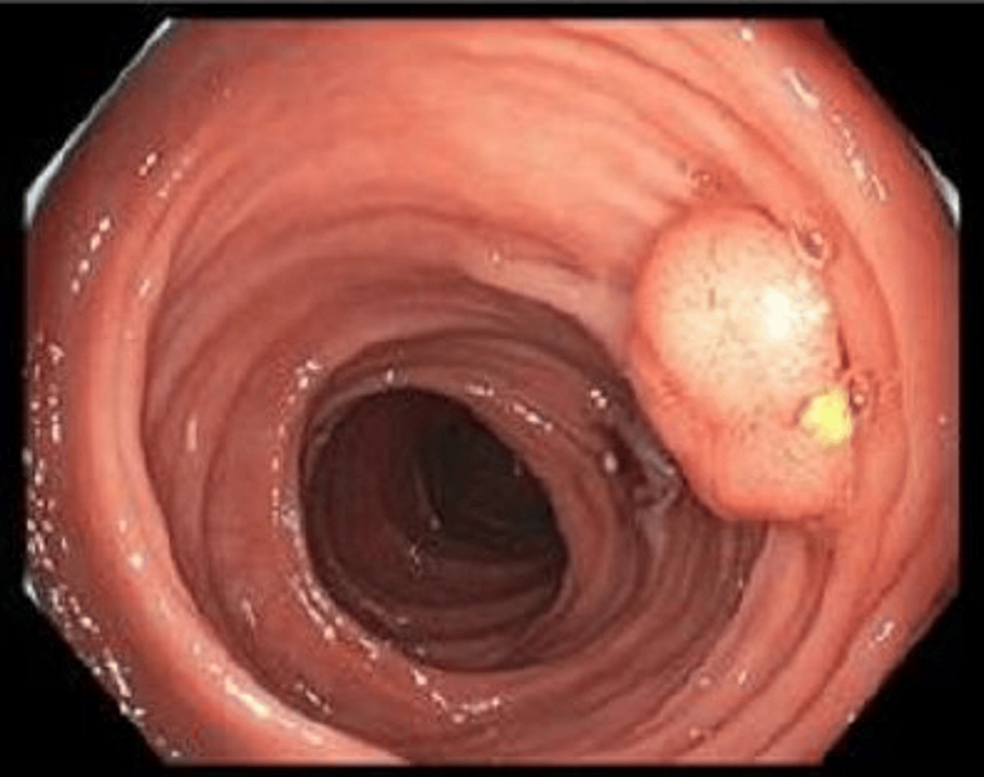
Sigmoid colon polyp seen on the patient's surveillance colonoscopy. Twelve total polyps were seen on the colonoscopy, the largest 12 mm.

A 52-year-old man with hypertension, hyperlipidemia, and a history of mixed-germ cell testicular cancer (1999) status post-right-sided orchiectomy and chemotherapy with recurrence resulting in retroperitoneal lymph node dissection and autologous stem cell transplant (2003) in remission presented with new-onset black tarry stools and new-onset solid-food dysphagia for two weeks. He denied nausea, vomiting, diarrhea, unintentional weight loss, fevers, night sweats, or sick contacts. He reported 200 mg ibuprofen use multiple times a day for one week after a right-hand injury. Other home medications included atorvastatin, baclofen, iron supplements, lisinopril, and tadalafil. His colonoscopy eight months prior revealed multiple sessile serrated and tubular adenomatous polyps, the largest one being 12 mm (Figure 1). He had a 20-pack-year history and previous alcohol use disorder but remained sober for more than three years.

**Figure 2:**
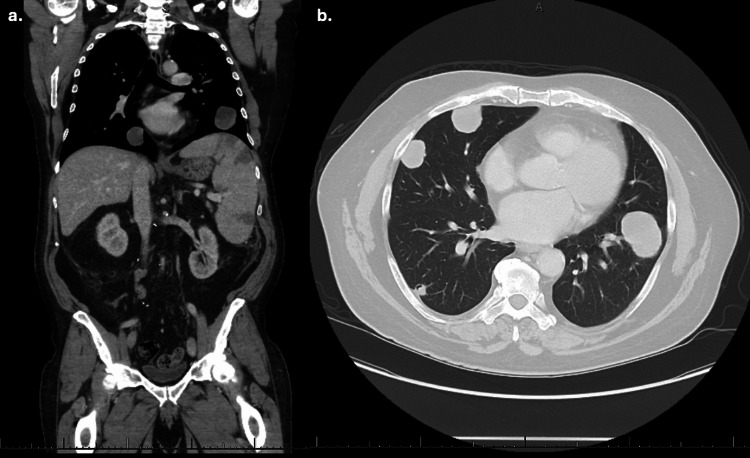
CT chest, abdomen, and pelvis with intravenous contrast in the coronal (a) and transverse (b) plain showing paraesophageal nodules, multiple pulmonary nodules, and splenomegaly with new splenic infarcts.

**Figure 3:**
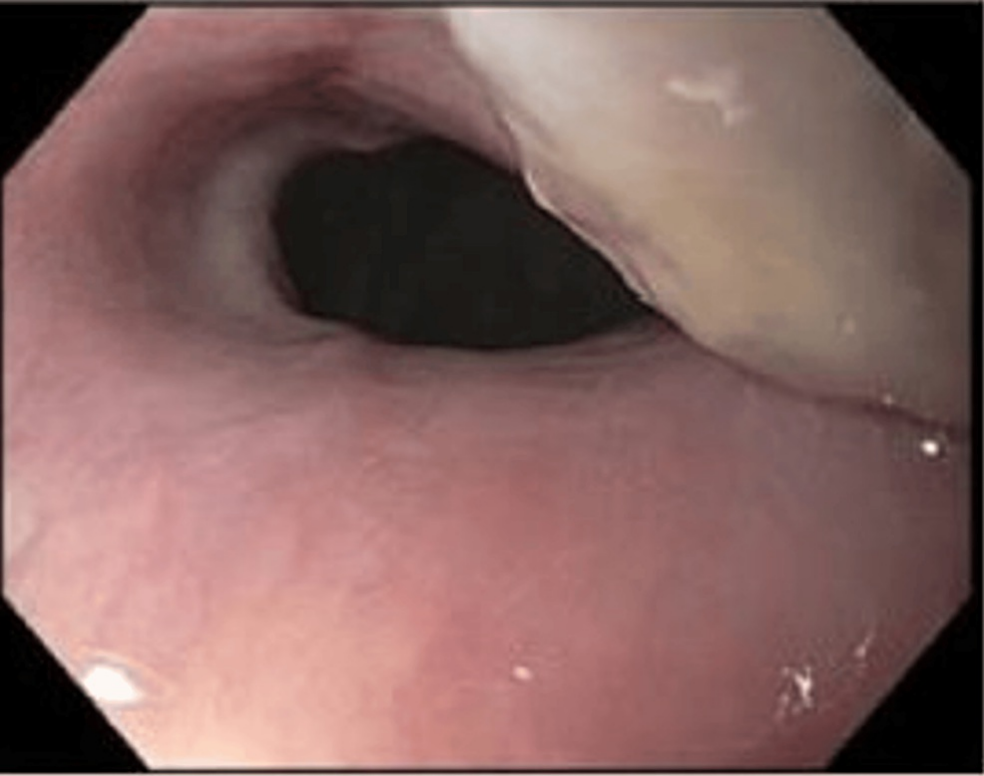
Partially ulcerated region in the esophagus at 30 cm with extrinsic compression.

**Figure 4:**
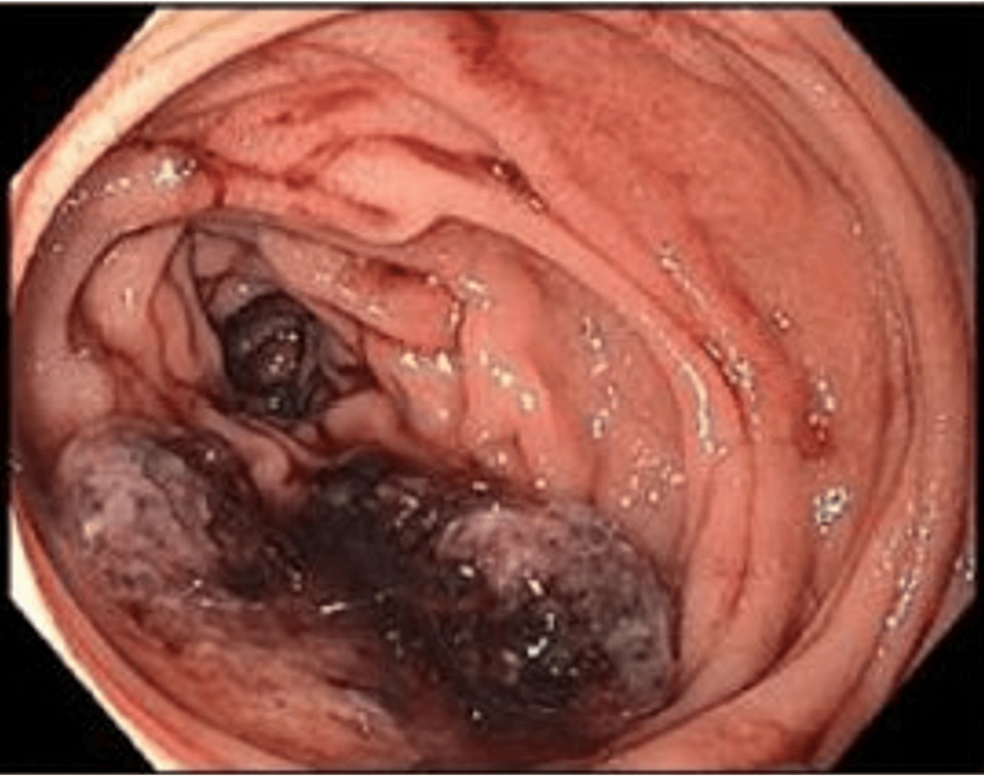
A non-obstructive, ulcerated, friable mass in the second portion of the duodenum.

There is no family history of gastrointestinal (GI) or hepatic cancer. Oncology surveillance computer tomography (CT) imaging was negative for recurrence, and the serum alpha-fetoprotein (AFP) level was undetectable two years prior to presentation. A physical exam was significant for melena. Labs were notable for beta-human chorionic gonadotropin (β-HCG) greater than 31,000 (upper limit of the testing range), lactate dehydrogenase (LDH) 553, hemoglobin (Hbg) 8.8, platelet (PLT) 191, white blood cell (WBC) 6.7, iron saturation of 7%, iron level of 20, and ferritin 101. AFP and comprehensive metabolic panel (CMP) were unremarkable (Table 1). CT chest, abdominal, and pelvis showed innumerable pulmonary metastatic lesions, a large mediastinal mass externally compressing the esophagus, and hepatic lesions (Figure 2). Endoscopy revealed an ulcerated lesion in the upper one-third of the esophagus with extrinsic compression (Figure 3) and a large non-obstructive ulcerated mass in the proximal duodenum with distal clotted blood distal duodenum (Figure 4). Endoscopic biopsy was deferred due to active bleeding. Instead, a CT-guided liver biopsy was obtained and found to have a PCC. The patient was referred to oncology for staging and treatment. 

**Table 1:** Laboratory tests. BUN: blood urea nitrogen, β-HCG: beta-human chorionic gonadotropin, LDH: lactate dehydrogenase.

Test	Lab value	Reference range
White blood count	6.8 K/mcL	4-10 K/mcL
Hemoglobin	8.3 g/dL	14-17 g/dL
Hematocrit	25.2%	41-51%
Platelets	191 K/mcL	150-350 K/mcL
Serum iron	20 μg/dL	20-250
Iron saturation	7%	20%-50%
Ferritin	101 μg/L	15-200 μg/L
D-dimer	871 μg/mL	<0.5 μg/mL
Sodium	135 mmol/L	136-145 mmol/L
Potassium	3.7 mmol/L	3.5-5.0 mmol/L
Chloride	105 mmol/L	98-106 mmol/L
CO_2_	26 mmol/L	23-28 mmol/L
BUN	15 mg/dL	8-20 mg/dL
Creatinine	0.78 mg/dL	0.7-1.3 mg/dL
Calcium	8.6 mg/dL	9-10.5 mg/dL
Bilirubin total	1.2 mg/dL	0.3-1.2 mg/dL
Alkaline phosphatase	113 units/L	36-92 units/L
Alanine transaminase	27 units/L	0-35 units/L
Aspartate transaminase	23 units/L	0-35 units/L
Alpha-fetoprotein	0	0-20 μg/L
β-HCG	>31,000 IU/L	<5 mIU/mL
LDH	553 IU/L	140-280 IU/L

## Discussion

Metastatic PCC commonly presents with anorexia, malaise, weight loss, cough, cervical or supraclavicular lymphadenopathy, back pain, and side effects of central nervous system mass effects like seizures or headaches [3]. PCC sites of metastasis include the lungs, lymph nodes, bones, and brain, and hepatic metastases are the most common intra-abdominal sites [3,4]. Fewer than 5% of patients present with metastasis to the gastrointestinal (GI) system, and furthermore, small intestine metastasis is even rarer, as described in case series and literature review [5-8].

Some presenting symptoms of GI metastasis include nausea, vomiting, occult or massive GI bleeding, weight loss, fatigue, and pallor. Symptoms are a combination of compression from the tumor mass, friable vessels, and anemia from blood loss [6]. Choriocarcinoma tends to ulcerate, become necrotic, and bleed due to outgrowing its blood supply, which may be one of the presenting symptoms or in response to chemotherapy [7,9]. PCC intestinal metastases are a predictor of poor prognosis [6]. In comparison, PCC lung metastases are common and can be managed effectively with chemotherapy [10]. PCC brain metastases tend to have a poor prognosis due to the high risk of intratumoral hemorrhage and treatment challenges in achieving remission [11]. PCC liver metastasis is associated with a very poor prognosis without effective treatment, survival period measured in months [12]. This patient was urgently re-established with his oncologist for treatment evaluation, given his poor prognosis. Aggressive chemotherapy regimens often include cisplatin, etoposide, and bleomycin [13]. The proposed mechanisms of metastasis to the GI tract are a combination of direct tumor infiltration and hematogenous dissemination. The primary lesion is hypothesized to either hematogenously spread to the liver first and then infiltrate the greater omentum or infiltrate the greater omentum and spread hematogenously to the liver and GI tract, invading the muscular layer [6,7]. The route of GI tract metastasis is difficult to assess due to no prior imaging capturing the PCC and the patient being in stage 4 on presentation with multiple hepatic and GI tract lesions. 

GI metastasis should be considered regardless of whether localizing symptoms such as nausea, vomiting, or abdominal pain from obstruction or blood loss are present in patients with PCC. Additional research identifying PCC tumor gene risk factors is needed. Conventional imaging modalities such as CT abdomen and pelvis used in this case may not identify gastrointestinal lesions otherwise present on endoscopic evaluation as seen in this case. Additionally, if the bleeding mass is in the distal jejunum or ileum, endoscopic intervention is limited. In this setting, angiography of the mesentery or erythrocyte scintigraphy is indicated [6]. Biopsy and pathological examination are the golden standards for the diagnosis of PCC, whether obtained endoscopically, surgically, or percutaneously under fluoroscopically [6]. 

## Conclusions

This case highlights an atypical presentation of metastatic PCC in a patient with new-onset melena and dysphagia with a previous history of mixed germ cell testicular cancer. PCC classically metastasizes to the lungs, lymph nodes, brain, and bones, while PCC metastasis to the GI tract is rare. This case highlights the aggressive nature of PCC and its potential to metastasize to unusual sites, such as the GI tract, necessitating early recognition for timely intervention and optimal management.
